# Leukocyte-Derived IFN-α/β and Epithelial IFN-λ Constitute a Compartmentalized Mucosal Defense System that Restricts Enteric Virus Infections

**DOI:** 10.1371/journal.ppat.1004782

**Published:** 2015-04-07

**Authors:** Tanel Mahlakõiv, Pedro Hernandez, Konrad Gronke, Andreas Diefenbach, Peter Staeheli

**Affiliations:** 1 Institute of Virology, University Medical Center Freiburg, Freiburg, Germany; 2 Spemann Graduate School of Biology and Medicine (SGBM), Albert Ludwigs University Freiburg, Freiburg, Germany; 3 Institute of Medical Microbiology and Hygiene, University Medical Center Freiburg, Freiburg, Germany; 4 International Max Planck Research School for Molecular and Cell Biology (IMPRS-MCB), Freiburg, Germany; 5 Research Centre Immunology and Institute of Medical Microbiology and Hygiene, University of Mainz Medical Centre, Mainz, Germany; Stanford University, UNITED STATES

## Abstract

Epithelial cells are a major port of entry for many viruses, but the molecular networks which protect barrier surfaces against viral infections are incompletely understood. Viral infections induce simultaneous production of type I (IFN-α/β) and type III (IFN-λ) interferons. All nucleated cells are believed to respond to IFN-α/β, whereas IFN-λ responses are largely confined to epithelial cells. We observed that intestinal epithelial cells, unlike hematopoietic cells of this organ, express only very low levels of functional IFN-α/β receptors. Accordingly, after oral infection of IFN-α/β receptor-deficient mice, human reovirus type 3 specifically infected cells in the lamina propria but, strikingly, did not productively replicate in gut epithelial cells. By contrast, reovirus replicated almost exclusively in gut epithelial cells of IFN-λ receptor-deficient mice, suggesting that the gut mucosa is equipped with a compartmentalized IFN system in which epithelial cells mainly respond to IFN-λ that they produce after viral infection, whereas other cells of the gut mostly rely on IFN-α/β for antiviral defense. In suckling mice with IFN-λ receptor deficiency, reovirus replicated in the gut epithelium and additionally infected epithelial cells lining the bile ducts, indicating that infants may use IFN-λ for the control of virus infections in various epithelia-rich tissues. Thus, IFN-λ should be regarded as an autonomous virus defense system of the gut mucosa and other epithelial barriers that may have evolved to avoid unnecessarily frequent triggering of the IFN-α/β system which would induce exacerbated inflammation.

## Author Summary

Virus-induced interferon consists of two distinct families of molecules, IFN-α/β and IFN-λ. IFN-α/β family members are key antiviral molecules that confer protection against a large number of viruses infecting a wide variety of cell types. By contrast, IFN-λ responses are largely confined to epithelial cells due to highly restricted expression of the cognate receptor. Interestingly, virus resistance of the gut epithelium is not dependent on IFN-α/β but rather relies on IFN-λ, questioning the prevailing view that receptors for IFN-α/β are expressed ubiquitously. Here we demonstrate that the IFN-α/β system is unable to compensate for IFN-λ deficiency during infections with epitheliotropic viruses because intestinal epithelial cells do not express functional receptors for IFN-α/β. We further demonstrate that virus-infected intestinal epithelial cells are potent producers of IFN-λ, indicating that the gut mucosa possesses a compartmentalized IFN system in which epithelial cells predominantly respond to IFN-λ, whereas other cells of the gut mainly rely on IFN-α/β for antiviral defense. We suggest that IFN-λ may have evolved as an autonomous virus defense system of the gut mucosa to avoid unnecessarily frequent triggering of the IFN-α/β system which, due to its potent activity on immune cells, would induce exacerbated inflammation.

## Introduction

The intestine has to maintain tolerance to the symbiotic gastrointestinal microflora, while mounting an effective immune response when challenged with opportunistic bacteria or enteric viruses. Thus, the intestinal mucosa, composed of the lining epithelium and underlying lamina propria cells, forms the first line of defense against pathogenic microorganisms entering the body via the oral route.

The type I interferon family (IFN-α/β) represents a key element of the innate antiviral defense [1–5]. In humans the type I IFN family encompasses 13 IFN-α, a single IFN-β and a few minor IFN subtypes (IFN-κ/ε/ω) that all bind to a single heterodimeric cell surface complex known as IFN-α/β receptor [6]. IFN-α/β receptor engagement activates the Jak-STAT signaling pathway and induces the expression of several hundred IFN-stimulated genes (ISGs), many of which exhibit direct antiviral activity [7–10]. In 2003, the type III IFN family (IFN-λ), encompassing 3 similar IFN-λ molecules, was discovered [11,12]. It quickly became clear that the induction and mechanism of action of IFN-λ and type I IFN are very similar [13–16], although IFN-λ uses a distinct receptor for signaling. These observations raised the question why two seemingly redundant antiviral systems may have evolved.

The major difference between the IFN-α/β and the IFN-λ systems is that IFN-λ receptor expression is confined mostly to the mucosal epithelium, whereas the IFN-α/β receptor seemingly is ubiquitously expressed [13]. Accordingly, IFN-α/β receptor-deficient mice show enhanced susceptibility to a large panel of different viruses [2,4]. On the contrary, mice lacking functional IFN-λ receptors control systemic viral infections quite well and are only slightly more susceptible to respiratory viruses than wild-type mice [14,16]. Interestingly, mice deficient in both IFN-α/β and IFN-λ are extremely susceptible to various respiratory viruses, demonstrating redundancy of the two IFN systems in the lung that is rich in epithelial cells [14].

The importance of the type I IFN system for controlling enteric viral infections varies greatly depending on the challenge virus. For example, IFN-α/β plays an important role in restricting virus-induced disease after oral inoculation of mice with poliovirus or human reoviruses, but it is of moderate importance in restricting rotavirus that exhibits a high tropism for gut epithelial cells [17–23]. We recently demonstrated that the IFN-λ system is essential for efficient control of rotavirus replication in intestinal epithelial cells [23]. This finding was surprising, considering the fact that receptors for IFN-α/β are believed to be expressed on all nucleated cells and raised the question of why the IFN-α/β system was unable to compensate for IFN-λ deficiency in this case.

We demonstrate here that intestinal epithelial cells express only low levels of the two chains of the IFN-α/β receptor complex, have a low density of IFN-α/β receptors on the surface and, accordingly, respond only very poorly to stimulation with type I IFN. Interestingly, besides responding strongly to IFN-λ, intestinal epithelial cells also readily produced IFN-λ but not IFN-α or IFN-β in response to viral triggers, suggesting that IFN-λ functions as an autonomous antiviral defense mechanism in the gut epithelium that requires no assistance by type I IFN. Virus challenge experiments of mice lacking functional receptors for either IFN-α/β or IFN-λ confirmed the concept of a compartmentalized intestinal mucosal IFN system and highlighted the exceptionally dominant role of IFN-λ in antiviral protection of intestinal epithelial cells and bile ducts.

## Results

### Intestinal epithelial cells show low level IFN-α/β receptor expression and do not respond to type I IFN

**Fig 1:**
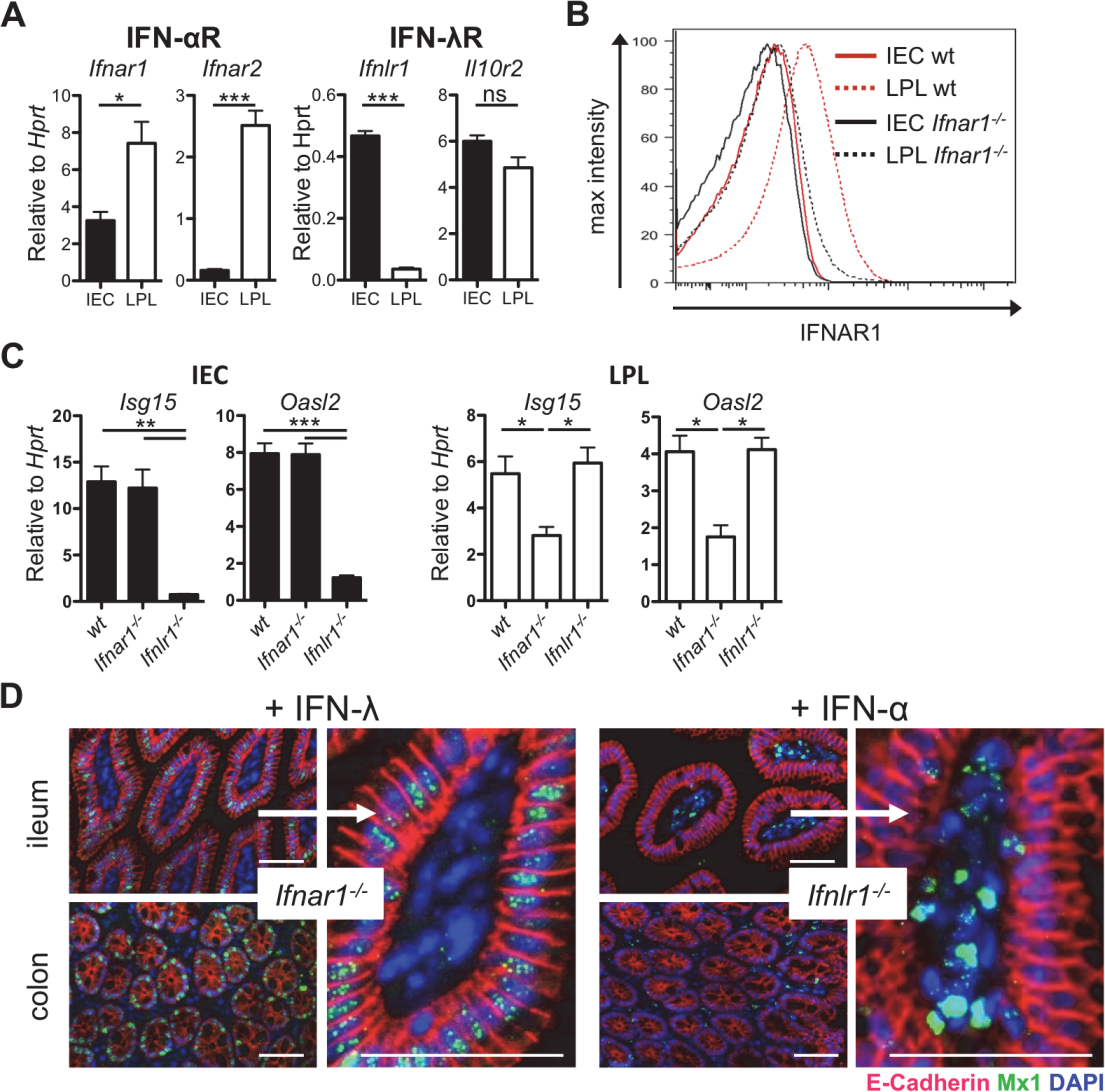
Intestinal epithelial cells minimally express IFN-α/β receptor and do not respond to type I IFN. **(A) RT-qPCR analysis of IFN-α/β receptor chains (*Ifnar1* and *Ifnar2*) and IFN-λ receptor chains (*Ifnlr1* and *Il10r2*) in intestinal epithelial cells (IEC) and lamina propria lymphocytes (LPL) isolated from whole intestinal tissue of adult wild-type mice (n = 4–8).** (B) IFNAR1 expression analyzed by flow cytometry on IEC or LPL fractions harvested from wild-type or *Ifnar1* ^*-/-*^ mice. (C) RT-qPCR analysis of two representative ISGs at steady state in IEC and LPL isolated from wild-type, *Ifnar1* ^*-/-*^ and *Ifnlr1* ^*-/-*^ mice (n = 3). (D) Adult *Ifnar1* ^*-/-*^ and *Ifnlr1* ^*-/-*^ mice were treated twice subcutaneously with 1 μg of mouse IFN-λ2 or human IFN-αB/D, respectively, at 24 h and 12 h prior to sacrifice as indicated. IFN-induced Mx1 in tissue sections from the gastrointestinal tract was visualized by immunofluorescence. IFN-responsive cells contain nuclear Mx1 (dotty structures in green). Epithelial cells express E-cadherin (red). DAPI (blue) stains nuclei. Data is representative for two to three independent experiments. Mean ± SEM. Bar = 100 μm. ns = non-significant, * p<0.05, ** p<0.01, *** p<0.001.

To address the question why the gut epithelium is poorly protected by type I IFN against infection with rotavirus [23], we measured IFN receptor gene expression in isolated intestinal epithelial cell (IEC) and lamina propria lymphocyte (LPL) fractions of adult wild-type mice by quantitative reverse transcription PCR (RT-qPCR). We analyzed the purity of isolated cell fractions by measuring the expression of marker genes of epithelial cells (*Cdh1* encoding E-cadherin) and leukocytes (*Ptprc* encoding CD45) (S1A and S2 Figs), as well as by flow cytometry using antibodies against CD45 and the epithelial marker EpCAM (S1B Fig). As expected, *Ifnlr1* and *Il10r2*, coding for the two chains of the IFN-λ receptor complex, were highly expressed in IECs but not in LPLs (Figs 1A and S2). Both components of the type I IFN receptor complex, *Ifnar1* and *Ifnar2*, were highly expressed in LPL that are well known to readily respond to type I IFN. By contrast, in IECs, we observed only low expression of *Ifnar1* and *Ifnar2* (Figs 1A and S2). Flow cytometric analysis revealed that the IEC fractions contained about 5% CD45^+^ cells (S1B Fig). Thus, we used fluorescence-activated cell sorting to purify epithelial (EpCAM^+^CD45^-^) cells from crude IEC and LPL fractions (S1B Fig). RT-qPCR analysis revealed strongly decreased expression of *Ifnar1 and Ifnar2* in purified epithelial cells when compared to leukocytes (EpCAM^-^CD45^+^) (S1C Fig).

To determine if low expression of *Ifnar1 and Ifnar2* in IECs would result in low levels of IFN-α receptor on the cell surface, we used an IFNAR1-specific antibody for immunostaining experiments. FACS analysis confirmed the presence of easily detectable levels of IFNAR1 on LPLs from wild-type but not *Ifnar1* ^*-/-*^ mice (Fig 1B). Importantly, under these experimental conditions, the staining of IECs from wild-type mice was not more intense than the staining of IECs from *Ifnar1* ^*-/-*^ mice (Fig 1B), demonstrating that cell surface expression of the type I IFN receptor on IECs is intrinsically low. These data offer a simple explanation for why type I IFN is anti-virally ineffective in intestinal epithelial cells.

Mononuclear phagocytes from germ-free mice do not readily mount type I IFN responses after TLR triggering or virus-mediated immune stimulation [24–26], suggesting that commensal bacteria-derived signals induce baseline IFN signaling that calibrates the activation threshold of various cell types and determines whether the host can mount a timely inflammatory response upon encountering a pathogen. We hypothesized that if IECs predominantly responded to IFN-λ, IECs of *Ifnar1* ^*-/-*^, but not *Ifnlr1* ^*-/-*^, mice should exhibit low steady state IFN responses. To evaluate this hypothesis, we measured the expression of two representative ISGs, *Isg15* and *Oasl2*, in IEC and LPL fractions isolated from intestinal tissue of wild-type, *Ifnar1* ^*-/-*^ and *Ifnlr1* ^*-/-*^ mice (Fig 1C). Indeed, baseline expression of ISGs was low in IECs of *Ifnlr1* ^*-/-*^ but not *Ifnar1* ^*-/-*^ mice. As expected, the reverse pattern of ISG expression was observed in LPLs, though the effect was less pronounced.

To comparatively analyze the roles of type I IFN and IFN-λ in inducing ISG expression in the intestinal mucosa, we treated mice subcutaneously with a high dose of recombinant IFN and stained tissues for IFN-induced Mx1 protein that accumulates in the nuclei of IFN-responsive cells. IFN receptor knockout mice were used to exclude background staining of Mx1 induced by endogenous IFN. Injection of IFN-λ into *Ifnar1* ^*-/-*^ mice led to a strong accumulation of Mx1 in the nuclei of E-cadherin-positive IECs of the gastrointestinal tract in its entire length (Figs 1D and S1D). In stark contrast, IFN-α injection into *Ifnlr1* ^*-/-*^ mice resulted in strong accumulation of Mx1 in the nuclei of lamina propria cells but not IECs (Figs 1D and S1D). Combined treatment with IFN-α and IFN-λ of *Ifnar1* ^*-/-*^ *Ifnlr1* ^*-/-*^ double knockout control mice did not result in detectable levels of Mx1 in the nuclei of either IECs or the cells of the lamina propria region (S1E Fig).

Type I IFN and IFN-λ have a redundant role in defense of the lung and upper respiratory epithelium against epitheliotropic respiratory viruses [14,27]. As a positive control for *in vivo* IFN treatment, we extracted tissues of the respiratory tract. Interestingly, a different picture emerged when tissue samples from the respiratory tract and the gut of the same animals were analyzed for the presence of Mx1. Similar to the gut mucosa, IFN-λ induced Mx1 exclusively in epithelial cells of respiratory tissues (S1F Fig). In IFN-α-treated animals, however, a broad range of cell types from the lung and trachea, including the E-cadherin-positive epithelial cells, showed prominent nuclear Mx1 staining (S1F Fig), indicating striking differences in type I IFN responsiveness in cells from the gut and the respiratory tract. No Mx1 staining was observed in tissue from the respiratory tract after combined treatment with IFN-α and IFN-λ of *Ifnar1* ^*-/-*^ *Ifnlr1* ^*-/-*^ double knockout control mice (S1G Fig). These results suggest that unlike epithelial cells of the lungs and the trachea, epithelial cells of the intestine rely almost exclusively on IFN-λ for antiviral defense.

### IECs are potent producers of IFN-λ

**Fig 2:**
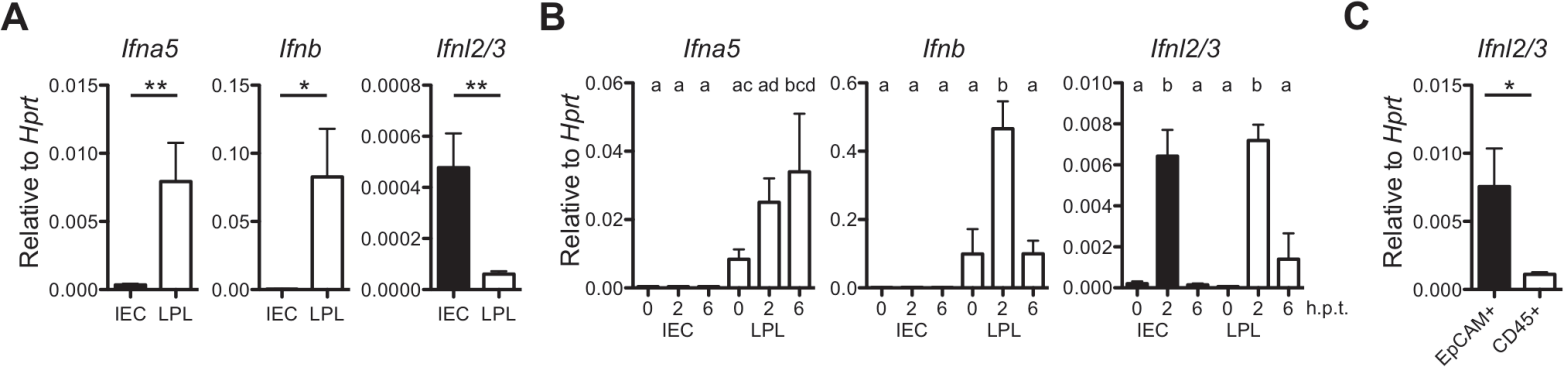
IECs are potent producers of IFN-λ but not type I IFN. (A) Base line expression of *Ifna5*, *Ifnb* and *Ifnl2/3* genes in IEC and LPL isolated from adult wild-type mice (n = 3) assessed by RT-qPCR. (B) Adult wild-type (n = 3) mice were injected intraperitoneally with 100 μg of polyI:C and intestinal tissue was harvested at 2 and 6 h post-treatment. Expression of *Ifna5*, *Ifnb* and *Ifnl2/3* was analyzed by RT-qPCR in IEC and LPL fractions. (C) Steady state IFN-λ gene expression analysis by RT-qPCR in FACS-sorted EpCAM^+^CD45^-^ and EpCAM^-^CD45^+^ cells from the epithelial fraction. Data is representative for two to three independent experiments. Mean ± SEM. * p<0.05, ** p<0.01. Different letters above bars mark significant differences (p<0.05).

Lymphocytes are crucial producers of type I IFN in the intestinal mucosa [17,28]. However, the IFN-λ-producing cell types in the gut have not been identified. Data shown in Fig 1C suggested constitutive production of baseline levels of IFNs in the gut. To identify the IFN-producing cells at steady state, we monitored type I IFN and IFN-λ gene expression by RT-qPCR in isolated IEC and LPL fractions from naïve adult wild-type mice. Substantial baseline expression of type I IFN genes was only observed in the LPL but not in the IEC fraction (Fig 2A). By contrast, baseline expression of IFN-λ genes was observed in the IEC fraction only (Fig 2A). Analysis of purified cells indicated that the IFN-λ-producing cells in the epithelial fraction express the CD45 marker (S3 Fig). Therefore, the IFN-λ-producing cells in the gut mucosa at steady state are of hematopoietic origin.

Dendritic cells are the major producers of IFN after virus infection or stimulation with the synthetic double-stranded RNA analogue poly I:C [29,30]. Indeed, intraperitoneal injection of poly I:C into wild-type mice triggered rapid expression of type I and type III IFN genes in cells of the LPL fraction. By contrast, the *Ifnl2/3* but not type I IFN genes were induced in cells of the IEC fraction under these conditions (Fig 2B). Interestingly, the cells strongly expressing IFN-λ in response to poly I:C in the epithelial fraction were not leukocytes but mainly EpCAM^+^ epithelial cells (Fig 2C). Collectively, these experiments identified IECs and a fraction of mucosal CD45^+^ immune cells as important sources of IFN-λ.

### IFN-λ restricts growth of reovirus in the intestinal epithelium and limits virus shedding in feces

**Fig 3:**
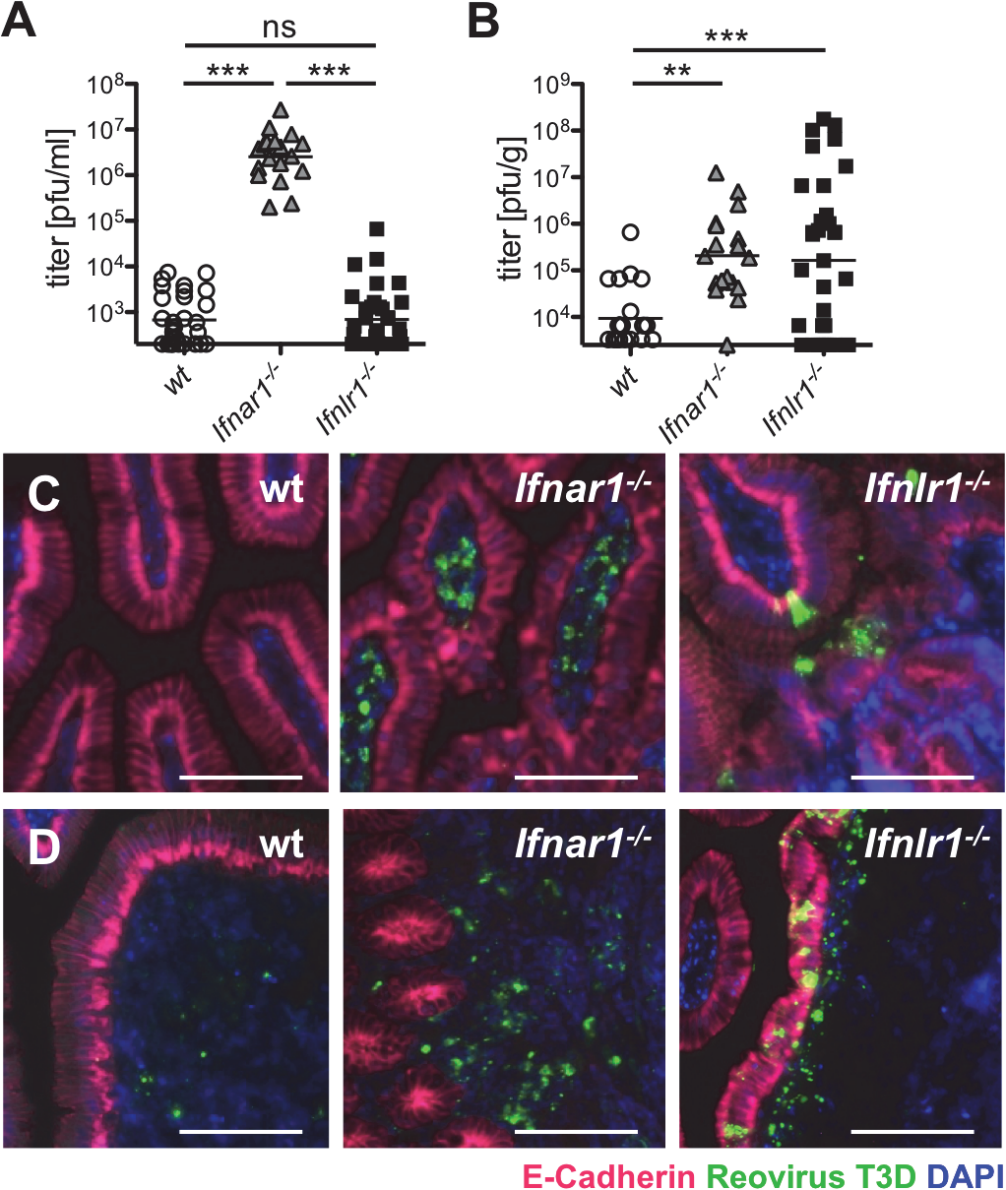
IFN-λ restricts reovirus replication in the epithelium and determines virus shedding in feces, whereas type I IFN blocks replication in the lamina propria and inhibits systemic dissemination. (A-D) Adult wild-type (n = 25), *Ifnar1* ^*-/-*^ (n = 18) and *Ifnlr1* ^*-/-*^ (n = 27) mice were inoculated intragastrically with 10^8^ pfu of reovirus T3D. Data pooled from several independent experiments. (A) At day 4 post-infection, reovirus replication in small intestinal tissue was analyzed by virus titration. (B) Reovirus titers in feces samples collected from wild-type and mutant mice at day 4 post-infection. (C, D) Immunostaining for reovirus antigen (green), E-cadherin (red) and DAPI (blue) in (C) small intestinal tissue or (D) Peyer’s patches. Images are representative of three independent experiments. Bar = 100 μm. ns = non-significant, ** p<0.01, *** p<0.001.

In earlier studies we used rotavirus to assess the role of IFN-λ in the gastrointestinal tract [23]. Due to the high tropism of rotaviruses for IECs, IFN-mediated antiviral effects in other cell types cannot be assessed with rotavirus. To determine why the IFN system is compartmentalized in the intestinal mucosa, we employed reovirus as a model [17,31–33]. Mammalian reoviruses have a wide cell tropism and exhibit a low degree of species specificity. After infection by the oral route, type I IFN signaling is of crucial importance for restricting systemic reovirus dissemination. By contrast, in IFN-competent hosts, reovirus-induced disease is mostly mild [17,34,35]. Indeed, intra-gastric inoculation of adult *Ifnar1* ^*-/-*^ mice with the human reovirus type 3 Dearing strain led to severe neurological symptoms such as hind limb paralysis, and all *Ifnar1* ^*-/-*^ animals had to be sacrificed by day 4 post-infection. By contrast, no symptoms were observed after infection of adult *Ifnlr1* ^*-/-*^ and wild-type mice (S4A Fig). Accordingly, reovirus replication in the terminal small intestinal tissue at day 4 post-infection was significantly higher in *Ifnar1* ^*-/-*^ mice compared to *Ifnlr1* ^*-/-*^ or wild-type animals (Figs 3A and S4B).

For efficient spread via the fecal-oral route, enteric viruses must be released from infected tissues to the gut lumen and excreted in feces. In Fig 1 we demonstrated that IFN-λ, but not type I IFN, induces a strong antiviral response in the epithelial barrier that separates the gut lumen from sterile host tissues. To examine the role of type I and type III IFNs in virus excretion, we quantified reovirus shedding in feces on day 4 post-infection. We observed high titers of infectious virus in feces of adult *Ifnar1* ^*-/-*^ and *Ifnlr1* ^*-/-*^ but not wild-type mice (Fig 3B). Considering the largely different virus titers in the intestinal tissue (Fig 3A), *Ifnar1* ^*-/-*^ mice excreted relatively small amounts of virus when compared to *Ifnlr1* ^*-/-*^ mice. Animals lacking both IFN receptors (*Ifnar1* ^*-/-*^ *Ifnlr1* ^*-/-*^) had comparable virus titers in the tissue to *Ifnar1* ^*-/-*^ mice, whereas virus shedding in feces was even higher than from *Ifnlr1* ^*-/-*^ mice (S4C Fig). Together, these results suggested that excreted virus may originate from different cell types in *Ifnar1* ^*-/-*^ and *Ifnlr1* ^*-/-*^ mice. Immunohistochemical staining of tissue samples from the small intestine confirmed this view. Reovirus antigen was almost exclusively found in IECs of *Ifnlr1* ^*-/-*^ mice at day 4 post-infection (Fig 3C, right panel), whereas reovirus was restricted to lamina propria cells in *Ifnar1* ^*-/-*^ mice (Fig 3C, middle panel). Reovirus specifically adheres to M cells and initially replicates in cells of the Peyer’s patch (PP) mucosa [36]. Accordingly, we detected reovirus antigen in cells of the PP of all three mouse strains (Fig 3D). In infected *Ifnar1* ^-/-^ mice the PPs were enlarged, and massive changes in the structure of the follicle-associated epithelium was observed (Fig 3D, middle panel) as previously described for reovirus type 1 [17]. In infected *Ifnlr1* ^*-/-*^ mice no such changes of the PP structure were noted. Interestingly, reovirus antigen in *Ifnlr1* ^*-/-*^ was detected in the follicle-associated epithelium as well as in the underlying tissue (Fig 3D, right panel). In contrast, reovirus staining in wild-type animals was restricted to few PP cells located under the epithelium (Fig 3D, left panel). These data indicate that reovirus produced by IECs of *Ifnlr1* ^*-/-*^ mice may more easily reach the feces than virus produced by cells in the tissue below, a mechanism accounting for the observed enhanced virus shedding in feces in *Ifnlr1* ^*-/-*^ mice.

### Reovirus replicates in epithelial cells of intestine and biliary tract, and induces fatal liver disease in suckling mice lacking functional IFN-λ receptors

**Fig 4:**
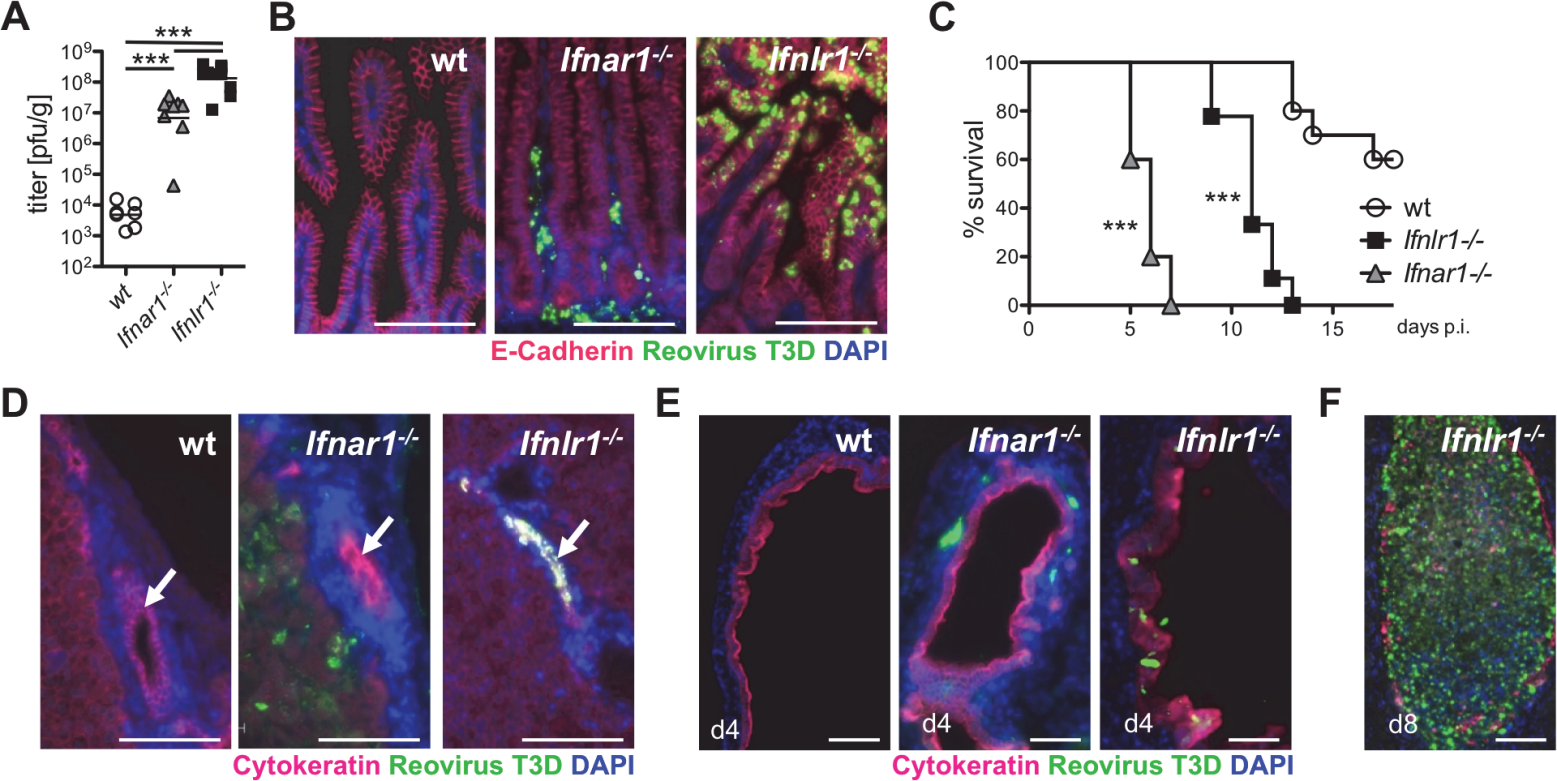
Reovirus replicates extensively in epithelial cells of intestine and biliary tract and induces fatal liver disease in suckling mice lacking functional IFN-λ receptors. Suckling wild-type (n = 7), *Ifnar1* ^*-/-*^ (n = 8) and *Ifnlr1* ^*-/-*^ (n = 11) mice were infected orally with 5 x 10^6^ pfu of reovirus T3D. Data from two independent experiments were pooled. (A) Reovirus titers in the small intestine on day 4 post-infection. (B) Immunostaining of small intestinal tissue at day 4 post-infection for reovirus antigen (green), E-cadherin (red) and DAPI (blue). (C) Survival kinetics of wild-type (n = 11), *Ifnar1* ^*-/-*^ (n = 13) and *Ifnlr1* ^*-/-*^ (n = 15) mice. (D) Immunostaining of liver tissue harvested on day 4 post-infection. Reovirus antigen (green), cytokeratin (red) and DAPI (blue). Arrows point to intrahepatic bile ducts. (E) Immunostaining of extrahepatic bile ducts for reovirus antigen (green), cytokeratin (red) and DAPI (blue) at day 4 post-infection. (F) Immunostaining for reovirus antigen of an extrahepatic bile duct from a diseased *Ifnlr1* ^*-/-*^ mouse on day 8 post-infection. Note that the duct is filled with material seemingly originating from virus-infected cells. Bar = 100 μm. ** p<0.01, *** p<0.001.

Severe viral gastroenteritis is a major problem of newborns and small children. Similarly, suckling mice are far more susceptible to rota- and reovirus-induced disease than adult animals [37–40]. We therefore asked whether the compartmentalized IFN response pattern, unique for the intestinal mucosa, might have more pronounced consequences for young animals. Two-days-old wild-type, *Ifnlr1* ^*-/-*^ and *Ifnar1* ^*-/-*^ animals were orally infected with reovirus, and viral titers in small intestine and colon were analyzed on day 4 post-infection. Contrary to the situation in adult mice, reovirus grew extremely well in the gut tissue of suckling *Ifnlr1* ^*-/-*^ mice, and virus titers in the small intestine (Fig 4A) and colon (S5A Fig) of such animals were significantly higher than in *Ifnar1* ^*-/-*^ mice. Immunohistochemistry revealed heavy infection of IECs in the terminal small intestine (Fig 4B) and colon (S5B Fig) of *Ifnlr1* ^*-/-*^ mice, whereas the virus was largely localized to the lamina propria region of the small intestine (Fig 4B) in *Ifnar1* ^*-/-*^ mice. Quantification of virus-infected cells demonstrated a striking differential cell tropism of reovirus to epithelial and non-epithelial cells in the small intestine depending on whether the receptors for IFN-α or IFN-λ were defective (S5C Fig). Low levels of infectious reovirus were measured in intestinal tissue of wild-type mice (Fig 4A), but no viral antigen was detected by immunostaining (Fig 4B).

Under these experimental conditions, *Ifnar1* ^*-/-*^ mice developed neurological symptoms and succumbed to reovirus infection within 5–7 days (Fig 4C). *Ifnlr1* ^*-/-*^ mice displayed no early neurological symptoms. However, on day 9 post-infection they started to develop symptoms typical for oily hair syndrome [40] accompanied by arrest of weight gain. By day 13, all reovirus-infected *Ifnlr1* ^*-/-*^ animals had died or had to be sacrificed due to severe disease (Fig 4C). The majority of reovirus-infected wild-type pups showed normal weight gain until day 11, when some animals started to develop neurological symptoms followed by a sudden weight loss and death over the next few days (Fig 4C).

After oral infection of suckling mice, reovirus type 3 may spread to the intrahepatic biliary epithelium (cholangiocytes) and to the brain, causing either liver disease or lethal encephalitis [40–43]. To determine whether the oily hair syndrome, observed in infected *Ifnlr1* ^*-/-*^ mice, was due to preferential replication of reovirus in biliary epithelial cells, we stained the liver and extrahepatic biliary tubules for virus antigen at day 4 post-infection. In these animals, large amount of reovirus antigen was indeed detected in extra- and intrahepatic cytokeratin-positive biliary epithelial cells, but seemingly not in any other cell type (Fig 4D and 4E). By contrast, reovirus-positive cells were present throughout the liver and detected adjacent to epithelial cells of the extrahepatic bile ducts of *Ifnar1* ^*-/-*^ mice, whereas the epithelium was largely virus free (Fig 4D and 4E). No virus antigen-positive cells were present in either the liver or the bile ducts of wild-type mice (Fig 4D and 4E). Analysis of tissues from *Ifnlr1* ^*-/-*^ mice that succumbed to the disease revealed destruction of the biliary epithelium, accumulation of virus antigen and blockade of the bile ducts (Fig 4F). H&E staining of the liver tissue on day 4 post-infection revealed inflammatory cells in all reovirus-infected animals with infiltrates localized predominantly around intrahepatic bile ducts in *Ifnlr1* ^*-/-*^ mice (S5D Fig). Taken together, these infection studies demonstrated a clear functional separation of the type I IFN and the IFN-λ system in antiviral defense of the gastrointestinal mucosa. They further revealed that reovirus can specifically target cholangiocytes of *Ifnlr1* ^*-/-*^ mice which prominently express functional IFN-λ receptors [44].

### Timely IFN-λ production by epithelial cells drives rapid clearance of intestinal reovirus infection

**Fig 5:**
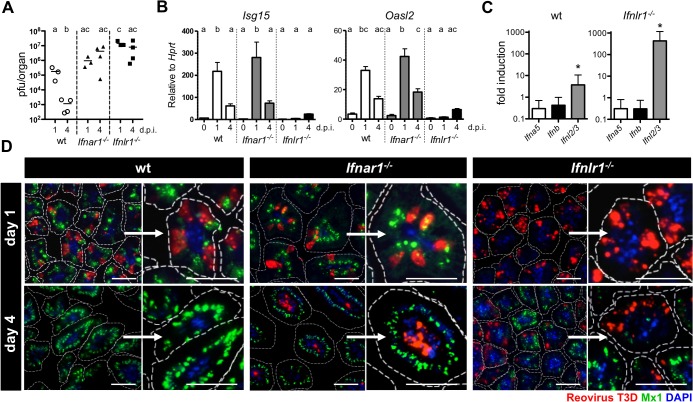
Timely IFN-λ production by epithelial cells drives rapid clearance of intestinal reovirus infection. Suckling wild-type, *Ifnar1* ^*-/-*^ and *Ifnlr1* ^*-/-*^ mice (n = 3–4) were orally infected with 5 x 10^6^ pfu of reovirus T3D, and tissue was harvested at either day 1 or day 4 post-infection. (A) Kinetics of reovirus replication by titration. (B) Expression of IFN-responsive genes *Isg15* and *Oasl2* in whole intestinal tissue analyzed by RT-qPCR. (C) Expression of *Ifna5*, *Ifnb* and *Ifnl2/3* genes in the IEC fraction of wild-type and *Ifnlr1* ^*-/-*^ mice assessed by RT-qPCR at day 1 post-infection. (D) Immunostaining of small intestinal tissue for reovirus antigen (red), Mx1 (green) and DAPI (blue). Dotted line marks the border of villi. Bar = 50 μm. d.p.i. = days post infection. Data representative for two individual experiments are shown. Mean ± SEM. Different letters above bars mark significant differences (p<0.05). * p<0.05.

To characterize the mucosal IFN system in more detail, we analyzed reovirus replication in intestinal tissue of suckling mice at day 1 and day 4 post-infection. On day 1, high level reovirus was detected in both wild-type and mutant mice by titration and RT-qPCR. Virus was largely cleared in wild-type mice by day 4, but was still present in guts of *Ifnar1* ^*-/-*^ and *Ifnlr1* ^*-/-*^ mice (Figs 5A and S6A). RT-qPCR analysis of RNA isolated from the whole intestinal tissue revealed strong expression of the IFN-regulated genes *Isg15* and *Oasl2* in wild-type and *Ifnar1* ^*-/-*^ mice on day 1 (Fig 5B). By contrast, *Ifnlr1* ^*-/-*^ mice failed to mount a proper IFN response on day 1 and showed a strongly attenuated response on day 4 post-infection (Fig 5B). A similar picture emerged when isolated IEC fractions rather than whole tissues were analyzed (S6B Fig), indicating that the protective response in the gut is predominantly mediated by IFN-λ, which is produced quickly after virus infection.

To visualize the virus-induced IFN response in the intestinal mucosa in time and space, we immunostained tissue sections from infected suckling mice for reovirus antigen and Mx1 at 1 and 4 days post-infection. At day 1, a large number of virus-positive IECs were detectable in the terminal part of the small intestine of wild-type, *Ifnar1* ^*-/-*^ and *Ifnlr1* ^*-/-*^ mice (Fig 5D). High levels of nuclear Mx1 were detectable in the majority of IECs in tissue samples from wild-type and *Ifnar1* ^*-/-*^ animals already at day 1 post-infection. In contrast, no Mx1 signals were detected in tissue from *Ifnlr1* ^*-/-*^ mice at this time point (Fig 5D, right panels), confirming that early responses are due to IFN-λ in the epithelium. At the early time post-infection, lamina propria cells that readily respond to exogenous type I IFN (see Fig 1) did not contain detectable levels of Mx1 in wild-type or *Ifnlr1* ^*-/-*^, suggesting that type I IFN production was low. By day 4 post-infection, wild-type animals had largely cleared the virus, whereas the intestinal tissue of *Ifnar1* ^*-/-*^ and *Ifnlr1* ^*-/-*^ mice was still heavily positive for reovirus antigen. Interestingly, on day 4 post-infection the vast majority of infected cells in *Ifnar1* ^*-/-*^ mice were located in the lamina propria region and not the epithelium as on day 1, whereas virus antigen remained associated with IECs in *Ifnlr1* ^*-/-*^ mice (Fig 5D, right panels). Virus clearance from IECs of wild-type and *Ifnar1* ^*-/-*^ mice by day 4 correlated with a strong IFN-λ-induced antiviral state, visualized by strong epithelial Mx1 staining. In *Ifnlr1* ^*-/-*^ mice, however, only a small fraction of villi were positive for Mx1 at this late time point, indicating that IFN-λ-independent antiviral responses were induced in only a small fraction of IECs by day 4 (Fig 5D). These data, together with RT-qPCR analysis of ISG expression (Fig 5B), demonstrate that during enteric virus infection type I IFN cannot compensate for the loss of IFN-λ, leading to prolonged replication of the virus in epithelial cells.

Minimal Mx1 expression was detected in cells of the lamina propria region. Nevertheless, these cells remained protected from reovirus in wild-type and *Ifnlr1* ^*-/-*^ mice, suggesting that type I IFN production during reovirus infection was low but protective (Fig 5D). Type I IFN that restricts systemic reovirus spread and protects mice against lethal infection is produced by dendritic cells [17]. IFN-λ production in virus-infected gut has to date not been analyzed. Earlier observations suggested that cells derived from the gut epithelium are potent producers of IFN-λ (see Fig 2). We therefore directly measured the expression of IFN genes by RT-qPCR in IEC fractions isolated from mock- and reovirus-infected mice. *Ifnl2/3* but not type I IFN genes were significantly induced in cells from wild-type mice on day 1 post infection (Fig 5C). In IEC preparations from *Ifnlr1* ^*-/-*^ mice that are highly susceptible to reovirus infection, induction of the *Ifnl2/3* genes was nearly 100-fold higher than in infected wild-type mice, whereas type I IFN genes were not expressed at enhanced rates at this early time point (Fig 5C). Our data demonstrates that IFN-λ produced by cells of the gut epithelium drives the early antiviral response that largely clears the virus from epithelial cells by day 4. Type I IFN appears to play no major role at this early stage of the reovirus infection. It rather prevents systemic virus spread.

## Discussion

The major conclusion from the experiments described here is that the intestinal mucosa possesses a highly compartmentalized IFN system that acts in concert to restrict enteric virus replication, and that the gut epithelium represents a unique cell compartment in the organism that does not strongly rely on IFN-α/β for antiviral defense but rather uses IFN-λ. We found that IFN-α/β was unable to induce the expression of antiviral genes in the intestinal epithelium and failed to protect these cells from infection with an enteric virus due to low expression of the IFN-α/β receptor complex. In contrast, IFN-λ induced robust antiviral protection in IECs. IFN-λ but not IFN-α/β genes were expressed at low but detectable levels in the gut mucosa of uninfected animals, and the gut epithelium produced high amounts of IFN-λ but not IFN-α/β in response to treatment of mice with an IFN-inducing chemical or after reovirus infection. Thus, the intestinal epithelium and the lamina propria are two compartments of the gut that not only preferentially produce but also preferentially respond to different types of IFN.

Mice lacking a functional IFN-α/β receptor show high susceptibility to a large number of viruses, including some attenuated virus strains which fail to induce disease in wild-type animals [2,4,14]. Interestingly, however, rotaviruses that preferentially infect the intestinal epithelium, are only moderately restricted by signaling through the type I IFN receptor [18,21,23], a finding that can easily be explained by the observation described in this report that IECs express the IFN-α/β receptor at only very low level. Likewise, norovirus replication in the gut is restricted by IFN-λ, whereas type I IFN controls norovirus replication in extra-intestinal sites [45].

Recent experimental evidence indicates a role of IFN-λ in virus control at various epithelial surfaces others than the gut [15,44]. However, it appears as if the contribution of IFN-λ at these other sites, including the lung, is mostly inferior to that of type I IFN. The reovirus data presented here explain our earlier observations with murine rotavirus which had pointed toward a non-redundant role of IFN-λ in epithelial cells of the intestinal tract [23]. Because reovirus can vigorously replicate in the epithelium of IFN-λ receptor-deficient mice, it has easy access to the gut lumen and is excreted in feces at high levels. The comparatively low fecal shedding of reovirus observed in IFN-α/β receptor-deficient mice might be explained by a barrier function of the largely virus-free epithelial layer that physically separates virus-producing lamina propria cells from the gut lumen.

We previously reported a significant role for IFN-λ in fecal shedding of rotavirus by adult mice [23]. We further reported that the respiratory SARS-CoV can be detected in feces of mice lacking both IFN receptor systems, but not in mice that only lack receptors for IFN-α/β [14]. Consistently, the *Stat1* and *Ifnlr1* but not the *Ifnar1* genes were recently shown to limit fecal norovirus shedding in mice [45]. Collectively, these results point to a substantial importance of IFN-λ in restricting virus excretion. We assume that other cytokines such as IFN-γ might also contribute, but this was not investigated here. Since the fecal-oral route is the major mode for transmission of enteric human viruses, such as poliovirus, norovirus, rotavirus, hepatitis E and A viruses, it is tempting to speculate that IFN-λ helps limiting excretion of these important human pathogens.

Plasmacytoid dendritic cells are very potent producers of biologically active IFN-α/β and IFN-λ [17,46,47], but most other cell types are also able to express IFN genes upon virus infection. Several recent studies suggested that cells of epithelial origin, such as the respiratory epithelium, keratinocytes and hepatocytes are potent producers of IFN-λ in virus-infected hosts [48–55]. We found that both IECs and hematopoietic cells in the epithelium strongly expressed IFN-λ but not IFN-α/β genes quickly after stimulation with poly (I:C) and in response to reovirus infection. Thus, the mucosal epithelium has evolved mechanisms to specifically produce IFN-λ. As similar signaling pathways are believed to control the expression of IFN-α/β and IFN-λ genes [56], this observation is surprising and suggests that the induction of genes encoding IFN-α and IFN-β is specifically blocked in IECs by an unknown mechanism. In polarized intestinal epithelial cells peroxisome-bound MAVS may preferentially trigger expression of *IFN-λ* genes [57], offering a potential explanation for our observations.

Orally administered reovirus has a broad tissue tropism in IFN-α/β receptor-deficient mice, infecting hepatocytes, myocardiocytes and many other cells, eventually causing a fatal disease [17,34]. We found that suckling mice lacking functional receptors for IFN-λ showed a milder disease than IFN-α/β receptor-deficient mice, although virus titers in the gastrointestinal tract of the IFN-λ receptor-deficient animals were substantially higher. This can probably be explained by the fact that severe damage of the gut epithelium has no immediate lethal consequences. Interestingly, reovirus-infected suckling mice deficient in functional receptors for IFN-λ showed symptoms resembling biliary atresia [58], which included oily fur syndrome and liver inflammation. Biliary atresia is a rare disease affecting one in 10,000 infants with etiology and pathology largely unknown. Infection with various viruses, including reovirus type 3, has been proposed to be associated with the disease in children [59,60]. Infection of mice with certain strains of rota- and reoviruses can reproduce most features of the human disease [61,62]. We detected virus antigen in cholangiocytes of our IFN-λ receptor-deficient but not wild-type or IFN-α/β-deficient mice that were infected with reovirus at early age, strongly suggesting that IFN-λ plays a decisive role in defending the biliary tract against viruses. This conclusion is consistent with our recent finding that mouse cholangiocytes are readily responding to exogenous IFN-λ [44]. Based on the results of our mouse model system, it is conceivable that children with biliary atresia may have genetic defect in their IFN-λ system.

Considering the fact that most cell types in the body are protected by IFN-α/β, our finding that type I IFN plays a negligible role in the gut epithelium is intriguing. In this context it is important to note that IFN-α/β is a double-edged sword. Besides inducing and regulating innate and acquired immunity against pathogens and tumors, IFN-α/β can also induce excessive inflammation (reviewed in [63])[64]. Chronic virus infections, such as HIV or LCMV, can lead to lymphocyte dysfunction due to prolonged IFN signaling [20,65] or refractoriness to IFN stimulation in hepatocytes in the case of HCV [66]. Therefore, it is tempting to speculate that the gut epithelium, which is in constant contact with commensal bacteria, has lost the ability to produce and respond to IFN-α/β due to its potential negative effects.

## Materials and Methods

### Ethics statement

All mice used in the study were bred locally in our facility and handled in accordance with guidelines of the Federation for Laboratory Animal Science Associations (www.felasa.eu/recommendations) and the national animal welfare body (Gesellschaft für Versuchstierkunde; www.gv-solas.de/index.html). Animal experiments were performed in compliance with the German animal protection law (TierSchG) and approved by the local animal welfare committee of the University of Freiburg (permit G-12/93).

### Mice

The mouse strains used have been described earlier [16]. Briefly, B6.A2G-*Mx1* wild-type mice carry intact *Mx1* alleles (wild-type), B6.A2G-*Mx1-Ifnar1* ^*-/-*^ mice lack functional IFN-α/β receptors (*Ifnar1* ^*-/-*^), B6.A2G-*Mx1-Ifnlr1* ^*-/-*^ mice lack functional IFN-λ receptors (*Ifnlr1* ^*-/-*^), and B6.A2G-*Mx1-Ifnar1* ^*-/—*^ *Ifnlr1* ^*-/-*^ double-knockout mice (*Ifnar1* ^*-/-*^ *Ifnlr1* ^*-/-*^) lack functional receptors for both IFN-α/β and IFN-λ. Newborn mice weighing 1.5 to 2 g or young adult mice (6–8 weeks of age) were used for experiments.

### Cells and virus

Reovirus type 3 Dearing strain was propagated on mouse fibroblast cell line L929 maintained in DMEM medium supplemented with 10% FCS. Newborn mice were orally inoculated with 5 μl of cell culture supernatant containing 5x10^6^ pfu of virus. Adult mice were intra-gastrically inoculated with 100 μl of virus (corresponding to 10^8^ pfu) using a 22G gastric gavage needle. Viral titers from feces and tissue homogenates were determined by plaque assay on L929 cells. Briefly, tissue was homogenized in 800 μl of PBS and feces in 500 μl of PBS using the FastPrep apparatus (MP Biomedicals). The homogenates were treated with chloroform (10% final concentration), centrifuged briefly and serial dilutions of the aqueous supernatants were incubated on L929 cells at room temperature. After 1 h, the inoculum was removed and cells were covered with 1.5% AVICEL in 1x DMEM medium containing 0.1% BSA. After four days medium was removed, cells were fixed with 4% paraformaldehyde and plaques were visualized with 0.5% crystal violet.

### *In vivo* treatment

One μg of hybrid human IFN-αB/D [67] or mouse IFN-λ2 (IL-28A; PeproTech) were subcutaneously injected in 100 μl of PBS. 100 μg of poly I:C was injected intraperitoneally in 200 μl of PBS.

### Immunohistochemistry

Tissue was fixed in 4% paraformaldehyde at 4°C for 24 h and embedded in paraffin. Antigen retrieval on deparaffinized 5-μm tissue sections on glass slides was performed in 0.01 M sodium citrate buffer at 121°C for 10 min. Tissue was permeabilized in 0.05% Triton-X100 and blocked with 10% normal donkey serum (Jackson ImmunoResearch) for 1 h at room temperature. Sections were incubated overnight at 4°C with rabbit-anti-reovirus T3D antiserum (a generous gift from T. Dermody, Vanderbilt University), mouse-anti-Mx1 monoclonal antibody M149 [68], rabbit anti-Mx1 polyclonal antiserum (AP5) [40] or mouse monoclonal anti-pan cytokeratin (Sigma), followed by the appropriate AF555-, AF488-, Cy3-, or Cy5-conjugated secondary antibody (Molecular Probes, Jackson ImmunoResearch). E-cadherin was stained with AF647-conjugated monoclonal mouse anti-E-cadherin antibody (BD Bioscience Pharmingen). Slides were mounted in DAPI-containing Vectashield (Vector Laboratories).

### Quantification of reovirus-positive cells in infected tissues

Tissue sections were stained for reovirus antigen and, simultaneously, E-cadherin to identify epithelial cells. The numbers of reovirus-positive cells expressing or lacking the E-cadherin were assessed by evaluating virus antigen-reactive cells in 3 visual fields of gut sections derived from at least 3 individual *Ifnar1* ^*-/-*^ and *Ifnlr1* ^*-/-*^ mice.

### Isolation of IEC and LP leukocytes

Isolation of intestinal epithelial cells and lamina propria leukocytes was performed as described previously [69]. Briefly, the whole small intestine was harvested, cut open longitudinally and washed briefly in PBS. Dissociation of epithelial cells was performed by incubation at 37°C in HBSS containing 5 mM EDTA and 10 mM Hepes on a shaker for 20 min. The remaining tissue was cut into pieces of approximately 1 mm^2^ before enzymatic digestion with 5 U/ml dispase (BD), 0.5 mg/ml collagenase D (Roche) and 0.5 mg/ml DNaseA (Sigma-Aldrich). Lymphocyte enrichment was performed by Percoll (Sigma-Aldrich) gradient centrifugation. The purity of isolated IEC and LPL fractions was confirmed by analyzing expression of epithelial (*Cdh1*-encoding E-cadherin) and leukocyte (*Ptprc*-encoding CD45) marker genes.

### Flow cytometry and cell sorting

Single-cell suspensions of lamina propria lymphocytes were analyzed using a FACS Canto II flow cytometer and the FACS Diva software (BD Biosciences). For data analysis, FlowJo V9.2 software (TreeStar) was used. To analyze cell surface expression of the IFN α/β receptor 1 chain, PE-labeled monoclonal antibody MAR1-5A3 (eBioscience) was used.

For sorting of intestinal epithelial cells and lymphocytes, the small intestines were cut into pieces of 1 mm^2^ and subjected to enzymatic digestion as described above. After blocking of Fc receptors with CD16/CD32 antibodies, single-cell suspensions were incubated with fluorescent conjugated antibodies against CD45 and EpCAM. After washing, cells were incubated with 4',6-diamidino-2-phenylindole (DAPI) for exclusion of dead cells and sorted using a BD FACSAria III cell sorter (BD Biosciences).

### RNA extraction and RT-qPCR

RNA was isolated with Trizol reagent (Invitrogen) according to manufacturer’s instructions. One μg of RNA was reverse-transcribed using QuantiTect Reverse Transcription Kit (Qiagen). Real-time PCR was performed using the QuantiTect SYBR Green PCR Kit (Qiagen) or TaqMan Universal Master Mix with gene specific probes (Applied Biosystems) and run on an ABIPrism 7900 sequence detector (Applied Biosystems). Samples were normalized to the expression of Hprt. The following mouse specific TaqMan assays were used: Hprt: Mm00446968_m1, Ifnl2/3: Mm04204156_gH, Ifna5: Mm00833976_s1 (Life Technologies). The following primers were used for SYBR Green based assays: Ifnb: forward, 5’- TCAGAATGAGTGGTGGTTGC -3’, reverse, 5’- GACCTTTCAAATGCAGTAGATTC -3’, Isg15: forward, 5’- GAGCTAGAGCCTGCAGCAAT -3’, reverse, 5’- TTCTGGGCAATCTGCTTCTT -3; Oasl2: forward, 5’- GGATGCCTGGGAGAGAATCG -3’, reverse, 5’- TCGCCTGCTCTTCGAAACTG -3’; Ifnlr1: forward 5’- GGAACTGAAGTACCAGGTGGA -3’, reverse 5’- GCCATAGGGAGTGTCAGGAA -3’; Il10rb: forward, 5’- TCTCTTCCACAGCACCTGAA -3’, reverse, 5’- GAACACCTCGCCCTCCTC -3’; Ifnar1: forward, 5’- CATGTGTGCTTCCCACCACT -3’, reverse, 5’- TGGAATAGTTGCCCGAGTCC -3’; Ifnar2: forward, 5’- GACCTTCGGATAGCTGGTGG -3’, reverse, 5’- CTCATGATGTAGCCGTCCCC -3’; Ptprc: forward, 5’-GAACTAAAACACATCTGGGAAAAATTA-3’, reverse, 5’-GCTTTCATGGTTGTTTTCACC-3’; Cdh1: forward, 5’-CAGGTCTCCTCATGGCTTTGC-3’, reverse, 5’-CTTCCGAAAAGAAGGCTGTCC-3’.

### Statistical analysis

Testing for statistical significance was performed on log-transformed viral titers by Student’s unpaired t-test or One-way ANOVA and Bonferroni multiple comparison test using Prism 4 software (GraphPad Software).

## Supporting Information

S1 Fig Epithelial cells of the gastrointestinal tract do not respond to type I IFN. (A) RT-qPCR analysis of epithelial marker gene *Cdh1* (E-cadherin) and leukocyte marker gene *Ptprc* (CD45) in IEC and LPL fractions. The data is representative for all cell isolation experiments described in the text. (B) Gating strategy for EpCAM^+^ epithelial cells and CD45^+^ lymphoid cells used in all FACS experiments. (C) IFN receptor genes were analyzed by RT-qPCR in FACS-purified CD45^+^ lymphoid cells and EpCAM^+^ epithelial cells from either the IEC or LPL fractions as indicated. (D-G) Adult *Ifnar1* ^*-/-*^, *Ifnlr1* ^*-/-*^ and *Ifnar1* ^*-/-*^ *Ifnlr1* ^*-/-*^ mice were treated twice subcutaneously with 1 μg of mouse IFN-λ2 and/or human IFN-αB/D at 24 h and 12 h prior to sacrifice as indicated (n = 2). IFN-induced Mx1 in tissue sections was visualized by immunofluorescence. IFN-responsive cells contain nuclear Mx1 (dotty structures in green). (E) Lack of IFN-induced Mx1 expression in tissue sections from the gastrointestinal tract or the (G) respiratory tract of *Ifnar1* ^*-/-*^ *Ifnlr1* ^*-/-*^ double-knockout mice simultaneously treated with IFN-λ2 and human IFN-αB/D. (F) Respiratory tissue sections of animals shown in (D and Fig 1D) were analyzed for Mx1 expression. Data are representative for several independent experiments. Bar = 100 μm. Mean ± SEM. * p<0.05, ** p<0.01, *** p<0.001.
(TIFF)

S2 Fig IFN receptor analysis in different fractions of the small intestinal tissue. IFN receptor gene expression analysed by RT-qPCR in whole gut tissue or isolated IEC fraction, LPL fraction or the leftover (stroma) (n = 3–5). The letters above bars mark significant significances (p<0.05). Mean ± SEM.
(TIFF)

S3 Fig Hematopoietic cells in the IEC fraction produce IFN-λ at steady state. CD45^+^ lymphoid cells and EpCAM^+^ epithelial cells were purified from IEC and LPL fractions before IFN gene expression was analyzed by RT-qPCR (n = 3–5). Mean ± SEM. * p<0.05, ** p<0.01, *** p<0.001.
(TIFF)

S4 Fig Disease and reovirus replication in the gut are mainly controlled by type I IFN in adult mice. Adult wild-type, *Ifnar1* ^*-/-*^ and *Ifnlr1* ^*-/-*^ mice were infected intragastrically with 10^8^ pfu of reovirus T3D. (A) Survival kinetics of adult wild-type (n = 6), *Ifnar1* ^*-/-*^ (n = 5) and *Ifnlr1* ^*-/-*^ (n = 13) mice. Data were pooled from two independent experiments. d.p.i. = days post-infection. (B) At day 4 post-infection, reovirus replication in terminal small intestinal tissue was analyzed by RT-qPCR (n = 7–9). (C) Adult wild-type mice or mice lacking functional IFN receptors were inoculated intragastrically with 10^8^ pfu of reovirus T3D. At day 4 post-infection, reovirus replication in small intestinal tissue and shedding in feces was analyzed by virus titration. Data pooled from several independent experiments are shown. ns = non-significant, ** p<0.01, *** p<0.001
(TIFF)

S5 Fig IFN-λ restricts reovirus replication and protects from liver inflammation in suckling mice. Suckling wild-type (n = 7), *Ifnar1* ^*-/-*^ (n = 8) and *Ifnlr1* ^*-/-*^ (n = 11) mice were infected orally with 5 x 10^6^ pfu of reovirus T3D. Data pooled from several independent experiments. (A) Reovirus titers in the colon on day 4 post-infection. (B) Immunostaining of colon tissue at day 4 post-infection for reovirus antigen (green), E-cadherin (red) and DAPI (blue). (C) Quantification of reovirus-infected cells in E-cadherin-positive (E-cad^+^) and-negative (E-cad^-^) cells from *Ifnlr1* ^*-/-*^ and *Ifnar1* ^*-/-*^ mice. (D) H&E staining of liver tissue. Images are representative of several independent experiments. Bar = 100 μm. Mean ± SEM. * p<0.05, *** p<0.001.
(TIFF)

S6 Fig Epithelial cell responses to reovirus infection depend on IFN-λ receptor signaling. Suckling wild-type, *Ifnar1* ^*-/-*^ and *Ifnlr1* ^*-/-*^ mice (n = 3–4) were orally infected with 5 x 10^6^ pfu of reovirus T3D, and epithelial cells were isolated at either day 1 or day 4 post-infection. (A) Kinetics of reovirus replication by RT-qPCR. (B) Expression of IFN-responsive genes *Isg15* and *Oasl2* analyzed by RT-qPCR. The letters above bars mark significant significances (p<0.05). Mean ± SEM. d.p.i. = days post infection.
(TIFF)
