# Supplementary figures and images for: Leukocyte-Derived IFN-α/β and Epithelial IFN-λ Constitute a Compartmentalized Mucosal Defense System that Restricts Enteric Virus Infections
Source: PLoS Pathog. 2015 Apr 7;11(4):e1004782. doi: 10.1371/journal.ppat.1004782 (PMC4388470; doi:10.1371/journal.ppat.1004782)

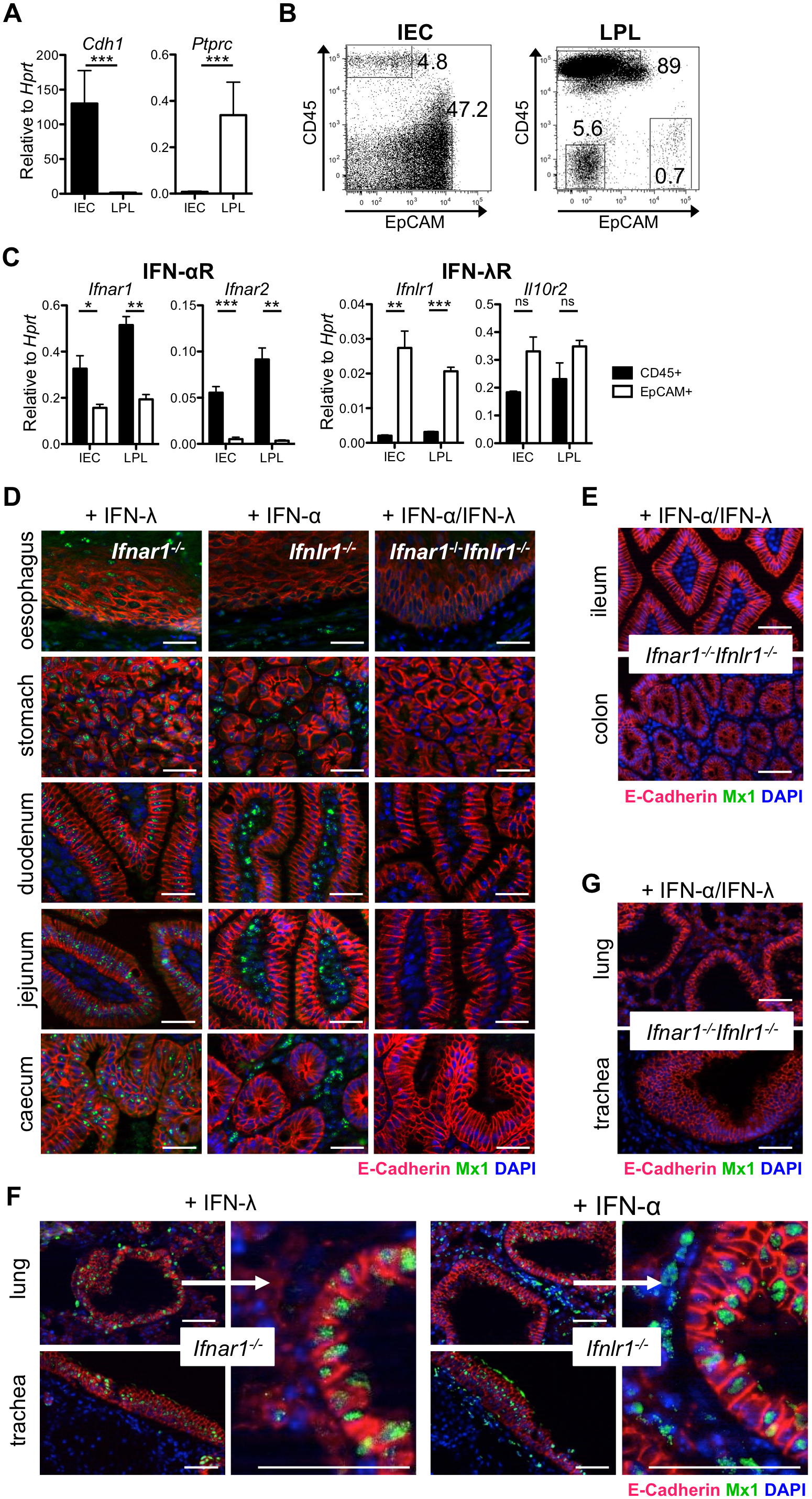

Supplement: S1 Fig — (A) RT-qPCR analysis of epithelial marker gene Cdh1 (E-cadherin) and leukocyte marker gene Ptprc (CD45) in IEC and LPL fractions. The data is representative for all cell isolation experiments described in the text. (B) Gating strategy for EpCAM+ epithelial cells and CD45+ lymphoid cells used in all FACS experiments. (C) IFN receptor genes were analyzed by RT-qPCR in FACS-purified CD45+ lymphoid cells and EpCAM+ epithelial cells from either the IEC or LPL fractions as indicated. (D-G) Adult Ifnar1 -/-, Ifnlr1 -/- and Ifnar1 -/- Ifnlr1 -/- mice were treated twice subcutaneously with 1 μg of mouse IFN-λ2 and/or human IFN-αB/D at 24 h and 12 h prior to sacrifice as indicated (n = 2). IFN-induced Mx1 in tissue sections was visualized by immunofluorescence. IFN-responsive cells contain nuclear Mx1 (dotty structures in green). (E) Lack of IFN-induced Mx1 expression in tissue sections from the gastrointestinal tract or the (G) respiratory tract of Ifnar1 -/- Ifnlr1 -/- double-knockout mice simultaneously treated with IFN-λ2 and human IFN-αB/D. (F) Respiratory tissue sections of animals shown in (D and Fig 1D) were analyzed for Mx1 expression. Data are representative for several independent experiments. Bar = 100 μm. Mean ± SEM. * p<0.05, ** p<0.01, *** p<0.001. (TIFF) [file ppat.1004782.s001.tiff]

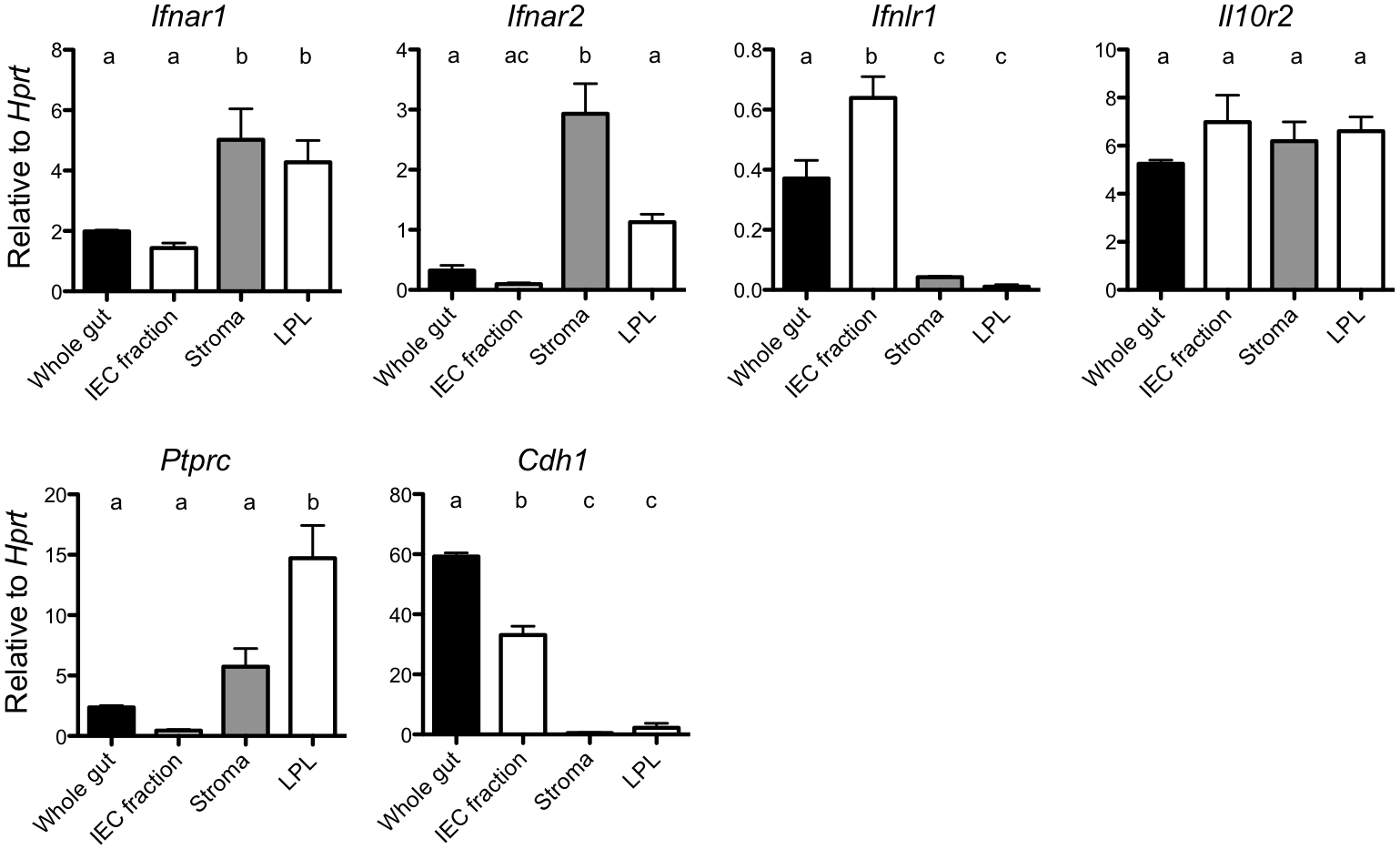

Supplement: S2 Fig — IFN receptor gene expression analysed by RT-qPCR in whole gut tissue or isolated IEC fraction, LPL fraction or the leftover (stroma) (n = 3–5). The letters above bars mark significant significances (p<0.05). Mean ± SEM. (TIFF) [file ppat.1004782.s002.tiff]

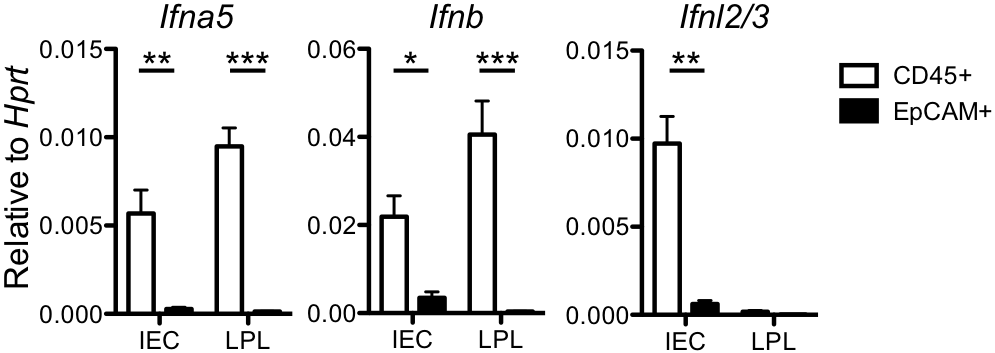

Supplement: S3 Fig — CD45+ lymphoid cells and EpCAM+ epithelial cells were purified from IEC and LPL fractions before IFN gene expression was analyzed by RT-qPCR (n = 3–5). Mean ± SEM. * p<0.05, ** p<0.01, *** p<0.001. (TIFF) [file ppat.1004782.s003.tiff]

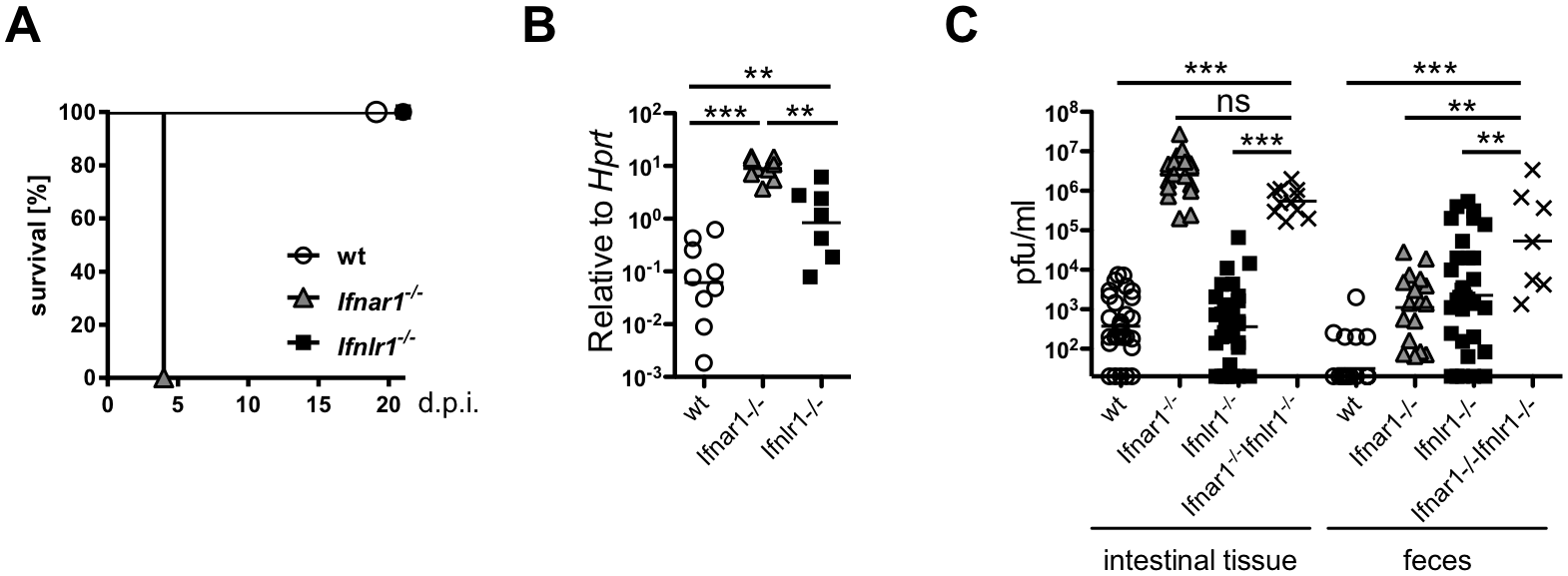

Supplement: S4 Fig — Adult wild-type, Ifnar1 -/- and Ifnlr1 -/- mice were infected intragastrically with 108 pfu of reovirus T3D. (A) Survival kinetics of adult wild-type (n = 6), Ifnar1 -/- (n = 5) and Ifnlr1 -/- (n = 13) mice. Data were pooled from two independent experiments. d.p.i. = days post-infection. (B) At day 4 post-infection, reovirus replication in terminal small intestinal tissue was analyzed by RT-qPCR (n = 7–9). (C) Adult wild-type mice or mice lacking functional IFN receptors were inoculated intragastrically with 108 pfu of reovirus T3D. At day 4 post-infection, reovirus replication in small intestinal tissue and shedding in feces was analyzed by virus titration. Data pooled from several independent experiments are shown. ns = non-significant, ** p<0.01, *** p<0.001 (TIFF) [file ppat.1004782.s004.tiff]

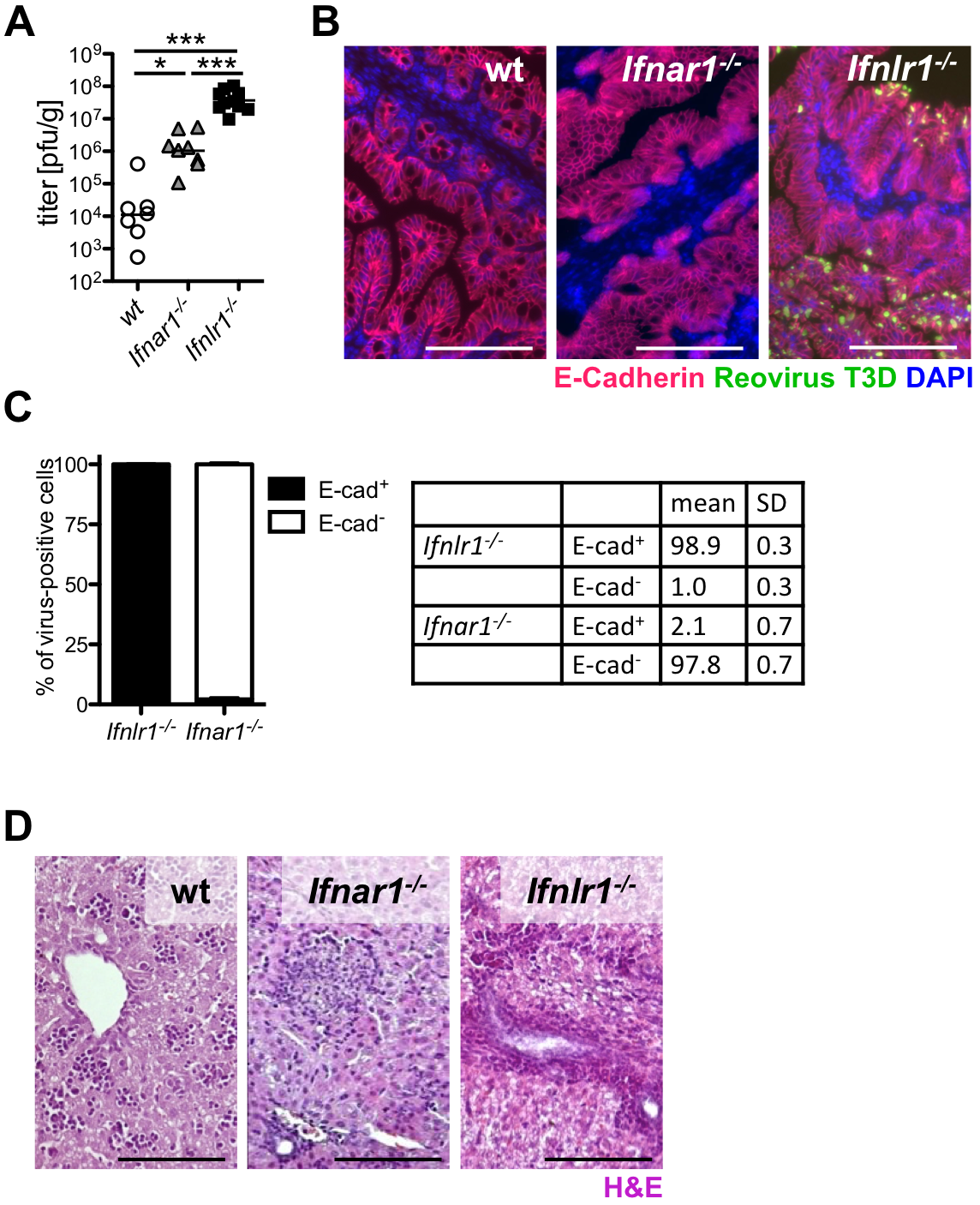

Supplement: S5 Fig — Suckling wild-type (n = 7), Ifnar1 -/- (n = 8) and Ifnlr1 -/- (n = 11) mice were infected orally with 5 x 10^6^ pfu of reovirus T3D. Data pooled from several independent experiments. (A) Reovirus titers in the colon on day 4 post-infection. (B) Immunostaining of colon tissue at day 4 post-infection for reovirus antigen (green), E-cadherin (red) and DAPI (blue). (C) Quantification of reovirus-infected cells in E-cadherin-positive (E-cad+) and-negative (E-cad-) cells from Ifnlr1 -/- and Ifnar1 -/- mice. (D) H&E staining of liver tissue. Images are representative of several independent experiments. Bar = 100 μm. Mean ± SEM. * p<0.05, *** p<0.001. (TIFF) [file ppat.1004782.s005.tiff]

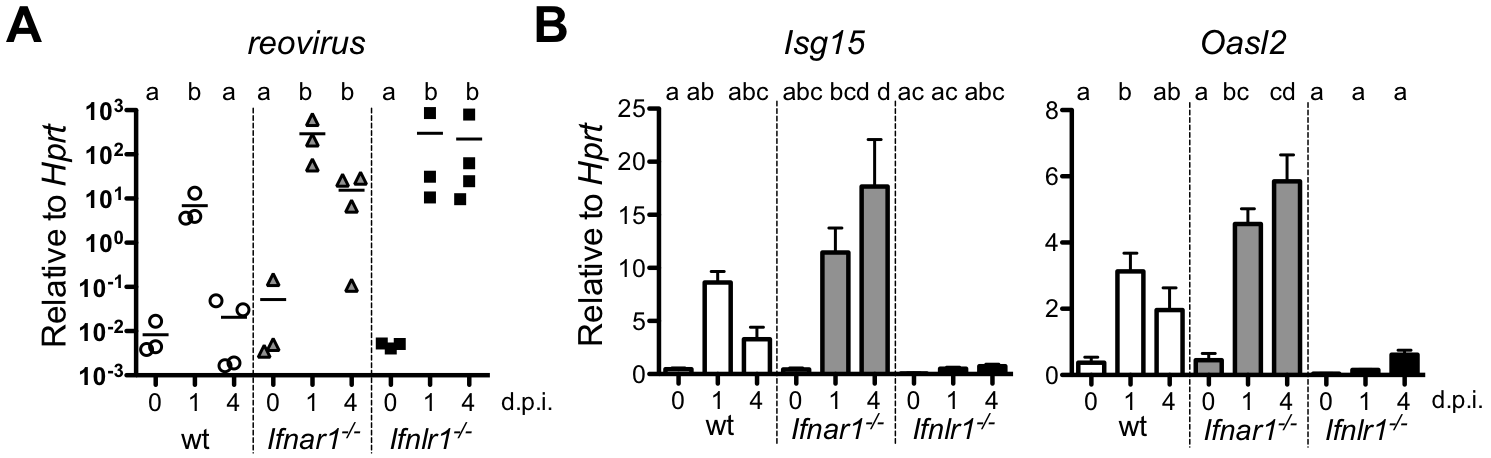

Supplement: S6 Fig — Suckling wild-type, Ifnar1 -/- and Ifnlr1 -/- mice (n = 3–4) were orally infected with 5 x 10^6^ pfu of reovirus T3D, and epithelial cells were isolated at either day 1 or day 4 post-infection. (A) Kinetics of reovirus replication by RT-qPCR. (B) Expression of IFN-responsive genes Isg15 and Oasl2 analyzed by RT-qPCR. The letters above bars mark significant significances (p<0.05). Mean ± SEM. d.p.i. = days post infection. (TIFF) [file ppat.1004782.s006.tiff]
